# First-principles study on structural, electronic, magnetic and thermodynamic properties of lithium ferrite LiFe_5_O_8_†

**DOI:** 10.1039/d2ra01656g

**Published:** 2022-05-26

**Authors:** Su-Yong Kim, Kwang-Su Kim, Un-Gi Jong, Chung-Jin Kang, Song-Chol Ri, Chol-Jun Yu

**Affiliations:** Chair of Computational Materials Design (CMD), Faculty of Materials Science, Kim Il Sung University P.O. Box 76 Pyongyang Democratic People’s Republic of Korea ug.jong@ryongnamsan.edu.kp cj.yu@ryongnamsan.edu.kp; Institute of Functional Materials, Faculty of Materials Science, Kim Il Sung University P.O. Box 76 Pyongyang Democratic People’s Republic of Korea

## Abstract

Lithium ferrite, LiFe_5_O_8_ (LFO), has attracted great attention for various applications, and there has been extensive experimental studies on its material properties and applications. However, no systematic theoretical study has yet been reported, so understanding of its material properties at the atomic scale is still required. In this work, we present a comprehensive investigation into the structural, electronic, magnetic and thermodynamic properties of LFO using first-principles calculations. We demonstrate that the ordered α-phase with ferrimagnetic spin configuration is energetically favourable among various crystalline phases with different magnetic configurations. By applying the DFT + *U* approach with *U* = 4 eV, we reproduce the lattice constant, band gap energy, and total magnetization in good agreement with experiments, emphasizing the importance of considering strong correlation and spin-polarization effects originating from the 3d states of Fe atoms. We calculated the phonon dispersions of LFO with ferrimagnetic and non-magnetic states, and subsequently evaluated the Gibbs free energy differences between the two states, plotting the *P*–*T* diagram for thermodynamic stability of the ferrimagnetic against non-magnetic state. From the *P*–*T* diagram, the Curie temperature is found to be ∼925 K at the normal condition and gradually increase with increasing pressure. Our calculations explain the experimental observations for material properties of LFO, providing a comprehensive understanding of the underlying mechanism and useful guidance for enhancing performance of LFO-based devices.

## Abstract 2

We systematically investigate the material properties of lithium ferrite LiFe_5_O_8_ – structural, magnetic, electronic, lattice vibrational properties and thermodynamic stability – using density functional theory calculations.

## Graphical abstract

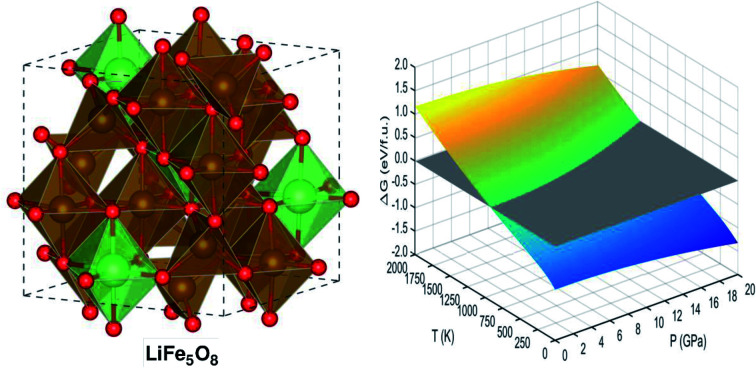

## 1 Introduction

Spinel-type ferrite materials, being metal oxides with ferromagnetism, have been studied and developed for several decades due to their excellent structural, electrical and magnetic properties that provoke various scientific and technological applications.^1–5^ Among the numerous ferrites, lithium ferrite LiFe_5_O_8_ (LFO) has attracted special interest in lots of technological applications, including as a cathode material in lithium-ion batteries,^6–8^ permanent magnets,^9,10^ spintronics^11^ and microwave devices.^12,13^ These LFO-based applications are associated with the peculiar electric and magnetic properties such as the high Curie temperature of ∼943 K, high saturation magnetization of 2.5 *μ*_B_ per formula unit, square-type hysteresis loop, low microwave dielectric loss, and high electric resistance.^14,15^

A lot of experimental work has been performed to manipulate the structural, electrical and magnetic properties for more useful applications of LFO. The most popular and efficient route in doing so is to make solid solutions by substituting suitable metal elements for Li or Fe in LFO.^16–18^ Many investigations have focused on Co-substituted LFO, reporting the significant impacts of Co substitution of the Fe cation on material properties of LFO.^8,19–23^ For instance, Sawant *et al.*^20,21^ reported the LFO-based solid solution Li_0.5_Co_*x*_Fe_2.5−*x*_O_4_ (0 < *x* < 0.6) where the Fe atoms were partly replaced with the Co atoms using the solution combustion method, demonstrating the varying material properties with varying Co content, *x*. They found a linear dependence of lattice constant on the Co content indicating the satisfaction of Vegard’s law, and almost uniform and regular morphology with large specific surface areas at all values of Co content. Most importantly, the resistivity and saturation magnetization were found to become larger while the dielectric loss lowers for higher Co content in Co-substituted LFO. In addition to the Co substitution, several investigations reported that the material properties can be effectively tuned by substituting Cu, Mn and Zn atoms for the Fe atom in LFO.^24–26^ Such substitutions can definitely lead to improvements in performance of the LFO-based devices, such as microwave and permanent magnets compared with the pure LFO.

On the other hand, the material properties of LFO have also been reported to be readily changeable according to the applied synthesis method. There have been different synthetic strategies developed for the preparation of lithium ferrite, such as conventional ceramic and double sintering method, and various wet-chemical methods including solution combustion, hydrothermal, and citrate precursor methods.^27–31^ In general, the conventional ceramic and double sintering methods require high processing temperatures of over 1200 °C, and because of the volatility of lithium above 1000 °C, such high processing temperature may lead to undesirable damage of electric and magnetic properties of LFO. Recently, Teixeira *et al.*^28^ synthesized the nanostructured lithium ferrite using the powdered coconut water-mediated sol–gel method, so-called the biogenic method, modulating the material properties using temperatures ranging from 400 to 1000 °C. They demonstrated that the high quality LFO crystal with higher dielectric constant, lower dielectric loss and smaller crystallite size could be obtained at lower temperatures (≤1000 °C) by this novel and eco-friendly biogenic method, when compared with the conventional ceramic method.

In spite of numerous experimental works for lithium ferrite LiFe_5_O_8_, a comprehensive understanding of its material properties has not yet been fully provided. To the best of our knowledge, there is only one theoretical study on the electronic and optical properties of LFO using density functional theory (DFT) calculations.^32^ Although some progress in understanding the microscopic origin of the electronic and optical properties has been made through theoretical work, Sousa *et al.*^32^ largely overestimated the band gap as *E*_g_ = 2.40 eV, while underestimating the dielectric constant as *ε* = 1.66, compared with the experimental values of *E*_g_ = 1.93 eV (ref. 33) and *ε* = 10 ∼ 14.^34^ This is found with no consideration of the strong correlation and spin-polarization effects of transition metal Fe atoms. Moreover, there is a lack of knowledge about the lattice vibrational properties and thermodynamic stability of LFO. Therefore, a comprehensive theoretical study on the underlying mechanism behind the structural, electronic and magnetic properties of LFO is necessary and important from both fundamental and practical points of view. In this work, we systematically investigate the material properties of lithium ferrite LiFe_5_O_8_ such as structural, magnetic, electronic, lattice vibrational properties and thermodynamic stability using the DFT calculations.

## 2 Computational methods

All the DFT calculations in this work have been performed by applying the pseudopotential plane-wave method as implemented in the Vienna *ab initio* simulation package (VASP).^35,36^ We employed the projector augmented wave (PAW) potentials^37,38^ provided in the package for a description of the Coulomb interaction between the ionic cores and the valence electrons. Here, the valence electronic configurations of atoms are Li-2s^1^, Fe-3d^7^4s^1^, and O-2s^2^2p^4^. We adopted the Perdew–Burke–Ernzerhof (PBE) functional^39^ within the generalized gradient approximation (GGA) for describing the exchange–correlation interaction among the valence electrons. Structural optimizations and electronic structure calculations were carried out with a plane-wave cutoff energy of 600 eV and *Γ*-centered *k*-point mesh of 8 × 8 × 8 for the conventional unit cell including 4 formula units (56 atoms). We performed variable cell structural optimizations until the atomic forces (pressures) became less than 10^−4^ eV Å^−1^ (0.003 GPa) with a total energy convergence threshold of 10^−8^ eV. These computational parameters guarantee a total energy accuracy of 2 meV per formula unit as shown in Fig. S11 in ESI.† For the calculations of electronic density of states (DOS), a denser *k*-point mesh of 12 × 12 × 12 was used, imposing partial occupancy on each orbital by the use of tetrahedron method with Blöchl correction.

In this work, we considered the on-site Coulomb interaction and spin polarization using DFT + *U* approach^5,40,41^ in order to take into account the strong correlation effect of Fe 3d states. For Fe atoms, various spin configurations were explored by applying the three different magnetic orderings – ferromagnetic (FOM), ferrimagnetic (FIM) and non-magnetic (NM). For the on-site Coulomb interaction parameter *U*, we repeated the structural optimizations and electronic structure calculations while varying the parameter *U* from 1 to 8 eV with a step of 1 eV, and then determined the reasonable value as *U* = 4 eV that gave the lattice constant and band gap with the best agreement with the experimental values.^28,42,43^ In addition, we calculated the frequency dependent dielectric constants, *ε*(*ω*) = *ε*_1_(*ω*) + *iε*_2_(*ω*), by applying density functional perturbation theory (DFPT).^44^

To proceed with the calculations of lattice vibrational and thermodynamic properties, we applied the finite displacement method using 2 × 1 × 1 supercells including 112 atoms, as implemented in the Phonopy code.^45^ For these supercell calculations, a reduced *k*-point mesh of 3 × 6 × 6 and cutoff energy of 400 eV were used in accordance with the larger size of supercell. The *q*-point mesh of 50 × 50 × 50 was used for the calculations of phonon DOS and thermodynamic potential functions such as Helmholtz and Gibbs free energies. The Gibbs free energy *G*(*T*,*P*) = *F*(*T*,*V*) + *PV* was given as a function of temperature *T* and pressure *P*, where *F*(*T*,*V*) is the Helmholtz free energy as a function of *T* and volume *V*. For evaluating the *PV* term, we calculated the DFT total energies *E* with systematically increasing volume from 0.95*V*_0_ to 1.05*V*_0_ with 11 intervals, where *V*_0_ is the equilibrium volume, and fitted the resultant *E*–*V* data into the empirical equation of state (EOS) for the solid,^46^ producing an *E*(*V*) function and therein pressure *P* using differentiation like *P* = −(∂*E*/∂*V*)_*T*_. Within the quasiharmonic approximation (QHA),^47–51^ the Helmholtz free energy *F*(*T*,*V*) was calculated by the following equation,1
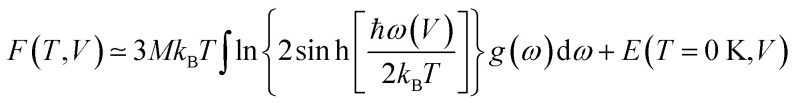
where *k*_B_ and ℏ are the Boltzmann and reduced Plank constants, respectively, and *ω*(*V*) is the phonon frequency as a function of volume *V*, *M* is the atomic mass, *g*(*ω*) is the normalized phonon DOS, and *E*(*T* = 0 K, *V*) is the DFT total energy as a function of *V* at 0 K. Finally, we estimated the thermodynamic stability of the FIM phase against the NM phase by calculating the Gibbs free energy difference,

2 Δ*G*(*T*,*P*) = *G*_FIM_(*T*,*P*) − *G*_NM_(*T*,*P*).

## 3 Results and discussion

### 3.1 Assessing crystalline structure, magnetic ordering and on-site parameters

**Fig. 1:**
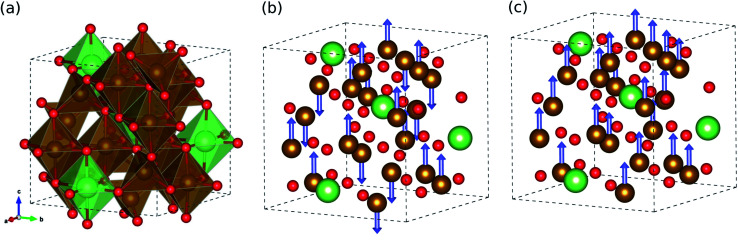
(a) Polyhedral view for crystalline structure of ordered α-phase LiFe_5_O_8_ with a space group of *P*4_3_32, and its ball-and-stick view in (b) ferrimagnetic and (c) ferromagnetic spin configurations. The brown, green and red balls represent the Fe, Li, and O atoms, respectively. The blue-coloured arrows indicate the spin direction of the Fe atom.

We started our work by determining the optimized crystalline structure and favourable spin configuration of Fe atoms in LFO. It is well accepted that LFO crystallizes in the completely inverse spinel structure with cubic symmetry, where the tetrahedral sites are filled by some of the Fe^3+^ cations and the octahedral sites are occupied by the remaining Fe^3+^ and Li^+^ cations.^30^ For crystallographic phases, there are two phases in LFO:^42^ one is the ordered α-phase with a space group of *P*4_3_32, where Li^+^ cations only occupy the octahedral 4b sites, and Fe^3+^ cations occupy the octahedral 12d sites and tetrahedral 8c sites, as shown in Fig. 1(a). The other phase is the disordered β-phase with a space group of *Fd*3*m*, where some of the Fe^3+^ cations locate at the tetrahedral 8a positions while the remaining Fe^3+^ and Li^+^ cations are randomly distributed over the 16d octahedral sites (see Fig. S1, ESI†). We reveal that the ordered α-phase is energetically lower than the disordered β-phase, with the best agreement of lattice constants to experiment^28^ (see Table S1, ESI†). This is consistent with a previous experiment,^43^ showing that the α-phase is stable below a temperature of ∼1008 K and the order–disorder phase transition occurs above this critical temperature.

With the ordered α-phase, we then determined the favourable spin configuration of the Fe atoms with the lowest total energy. For the FOM state, the Fe^3+^ cations at the octahedral 12d and tetrahedral 8c sites were considered to be arranged in parallel spin directions (Fig. 1(c)), whereas for the FIM state the Fe^3+^ cations at the octahedral sites (spin up) were supposed to have the opposite spin direction with those at the tetrahedral sites (spin down), as shown in Fig. 1(b). However for the NM state, the spin polarization effect of Fe atoms was not considered. It turns out that among the three different spin configurations, the FIM state was energetically favourable with a high total magnetization per formula unit of 2.5 *μ*_B_ f.u.^−1^ in good agreement with previous experiments^42,43^ (see Table S1, ESI†).

In the FIM state, although the spins of the Fe atoms at the octahedral and tetrahedral sites are arranged antiparallel, the net magnetization does not vanish because the octahedral site has 1.5 times more Fe atoms than the tetrahedral site. Such high magnetization leads to potential applications of lithium ferrite as permanent magnets and in various microwave devices. Meanwhile, the FOM state was found to have a much higher total magnetization of 6.5 *μ*_B_ f.u.^−1^ but with higher DFT total energy (∼3.7 eV f.u.^−1^) compared with the FIM state (see Table S1, ESI†). Based on these calculations for the crystalline structure and magnetic ordering, further calculations for the electronic and lattice vibrational properties considered only the ordered α-phase with ferrimagnetic ordering.

**Fig. 2:**
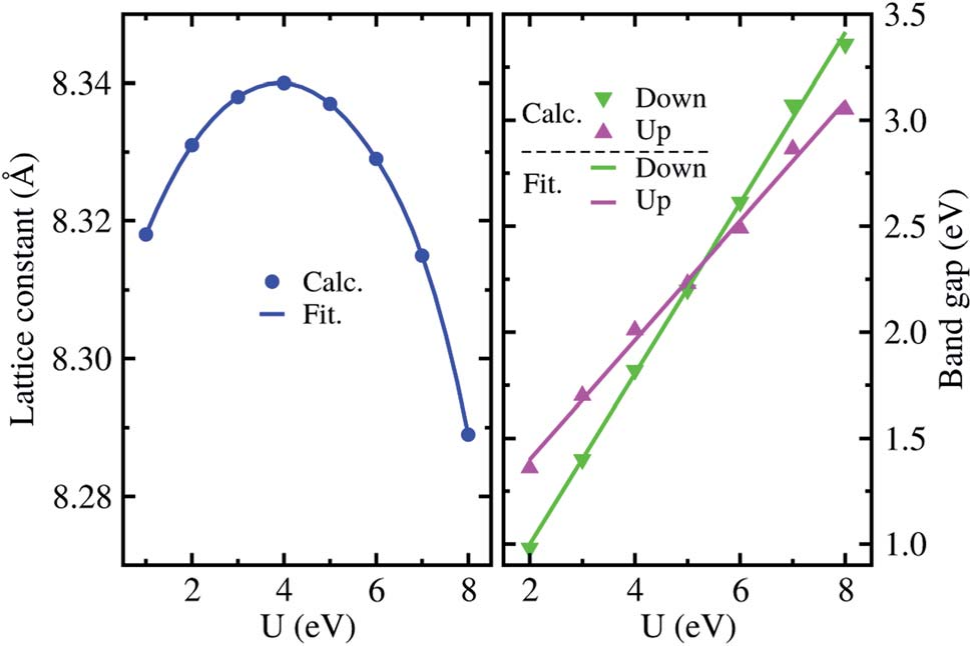
Lattice constants and band gaps for spin up and down states as a function of on-site Coulomb interaction parameter *U* in the ordered α-phase of LiFe_5_O_8_ with ferrimagnetic ordering. Calc. and Fit. mean the calculation and fitting, respectively.

Fig. 2 shows the lattice constants and band gaps calculated with gradually increasing values of the on-site Coulomb interaction parameter *U* from 1 eV to 8 eV. It was found that the lattice constant varies along the quadratic function while the band gap increases along the linear function of *U*. For the lattice constants, we obtained the fitting function of *a* = 8.297 + 0.023*U* − 0.003*U*^2^ (Å), finding the maximum value of 8.34 Å at the *U* = 4 eV in good agreement with the experimental value of 8.33 Å (ref. 28 and 43) with a very small relative error of 0.5%. For the band gap, the linear fitting functions were obtained to be *E*_g_ = 0.84 + 0.28*U* (eV) and *E*_g_ = 0.19 + 0.40*U* (eV) for the spin up and down state, respectively. Such increasing tendencies of band gaps are related to the conduction bands (CBs) pushing up while the valence bands (VBs) are pushed down with increasing on-site interaction parameter *U* (see Fig. S2, ESI†). At *U* = 4 eV, the band gap for the spin up state was calculated to be 1.99 eV, which agrees well with the experimental value of ∼1.93 eV.^33^ We note that the total magnetization of 2.5 *μ*_B_ f.u.^−1^ is independent of the value of *U* for the ordered α-phase. Accordingly, we fixed the on-site Coulomb interaction parameter to *U* = 4 eV for further calculations of electronic structure, lattice vibrational and thermodynamic properties.

### 3.2 Electronic structure

**Fig. 3:**
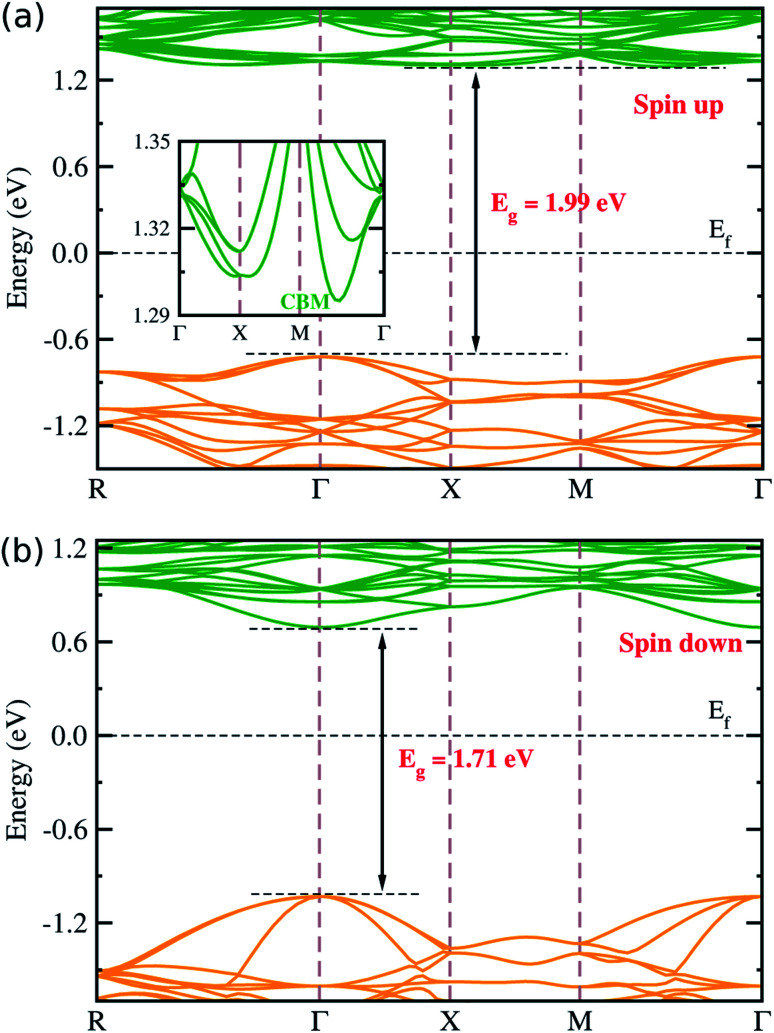
Electronic band structures for (a) spin up and (b) spin down states of the ordered α-phase LiFe_5_O_8_ with ferrimagnetic ordering, calculated by the DFT + *U* approach with *U* = 4 eV. The inset in (a) shows the detailed conduction band minimum (CBM) for the spin up state. Valence (conduction) bands are displayed by brown (green) lines. The Fermi level (*E*_f_) is set to zero.

Fig. 3 displays the electronic band structures for spin up and down states of the ferrimagnetic LFO, calculated by the DFT + *U* approach with *U* = 4 eV. The band structures were computed along the symmetry lines of *R* (0.5, 0.5, 0.5) → *Γ* (0.0, 0.0, 0.0) → *X* (0.5, 0.0, 0.0) → *M* (0.5, 0.5, 0.0) → *Γ* in the Brillouin Zone (BZ) according to the crystalline symmetry of LFO. It was found that the minority spin down states have a direct band gap of 1.71 eV at the *Γ* point, while the majority spin up states have an indirect band gap of 1.99 eV with the conduction band minimum (CBM) along the *M* → *Γ* line (Fig. 3(a) inset) and the valence band maximum (VBM) at the *Γ* point. In contrast, as mentioned above, Sousa *et al.*^32^ obtained an indirect band gap of 2.30 eV, being largely overestimated compared with the experimental value of 1.93 eV;^33^ using the modified Becke–Johnson potential^52^ but without considering the on-site Coulomb interaction and spin polarization. When considering only the spin polarization without on-site Coulomb interaction, LFO was found to be metallic with a zero band gap (see Fig. S3, ESI†), indicating that only PBE is not capable of correctly describing LFO. Therefore, we should emphasize that the DFT + *U* approach with *U* = 4 eV, considering both the spin polarization and on-site Coulomb interaction, could give a band gap and total magnetization in good agreement with experiments.^28,42,43^ It is worth noting that the band gap can be tuned by coherent phonon^53^ and pressure.^54^

**Fig. 4:**
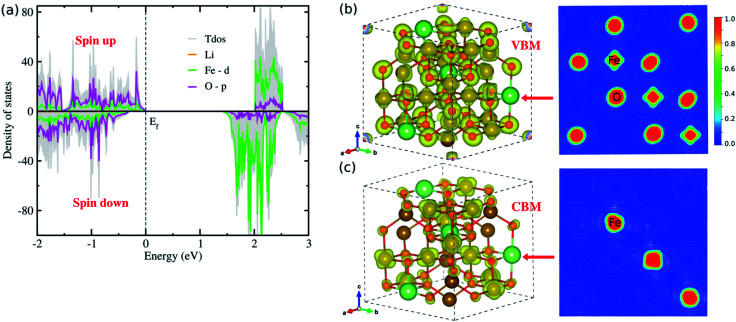
(a) Atom-projected density of states (DOS), where positive (negative) values represent spin up (down) states, (b) and (c) isosurface and contour plots of electronic charge densities corresponding to VBM and CBM, respectively, at a value of 0.02 |*e*| Å^−3^, where the contour plots are drawn in the crystal plane indicated by red arrows for the ordered α-phase of LiFe_5_O_8_.

To gain insight into the electric structure, we look deep into the atom-projected DOS and electronic charge densities corresponding to VBM and CBM, as shown in Fig. 4. Through the DOS analysis, it was found that the VBs are characterized by strong p–d hybridization between the O 2p and Fe 3d states, while the CBs are dominated by Fe 3d states (Fig. 4(a)). It should be noted that the Li atoms have negligible contribution to the upper VBs and lower CBs, and the Fe 3d (O 2p) states constitute the major contribution to the Fe (O) atoms (Fig. S4 and S5, ESI†). Moreover, we found that the total DOS for spin up (positive) and down (negative) states have inconsistent contributions, thereby offering non-zero net magnetization for the ferrimagnetic state of LFO. On the other hand, the p–d hybridization between the Fe and O atoms (major contributions of the Fe 3d states) in the VBs (CBs) was further confirmed from the analysis of the electronic charge densities corresponding to VBM (CBM) in Fig. 4(b and c). Based on these electronic structure calculations, we can expect that due to the clearly different behaviours between spin up and down states LFO can be used for spintronic applications. On the other hand, we showed the frequency-dependent dielectric constant *ε*(*ω*) (Fig. S6, ESI†), demonstrating that the *ε*(*ω*) reaches the maximum value of ∼10.8 in reasonable agreement with the experimental value of ∼13.1 (ref. 34) observed over a high frequency band of 3–30 MHz.

### 3.3 Lattice vibrational and thermodynamic properties

We next considered the lattice vibrational and thermodynamic properties of LFO. We obtained the phonon dispersions of LFO in the FIM and NM states, and evaluated the thermodynamic potential functions such as Gibbs and Helmholtz free energies at finite temperature and pressure. Then, the Gibbs free energy differences between the FIM and NM states were calculated using eqn (2) to assess the *P*–*T* diagram for thermodynamic stability of the FIM state against the NM state. From the *P*–*T* diagram, we obtained the Curie temperature *T*_C_ as a function of pressure, which is a key factor for determining the temperature range for a stable FIM state at a given pressure.

**Fig. 5:**
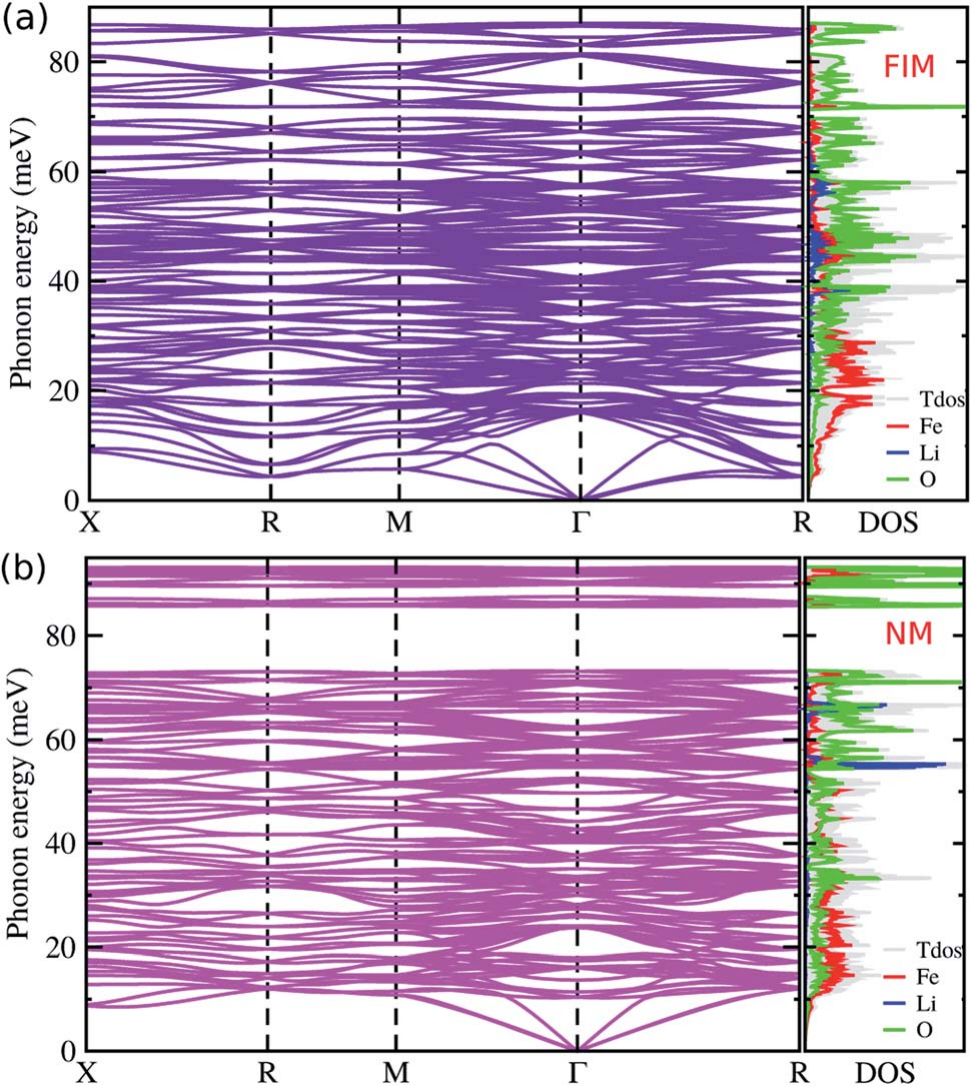
Phonon dispersion curves and the atom-projected phonon DOS of the ordered α-phase LiFe_5_O_8_ with (a) ferrimagnetic (FIM) and (b) non-magnetic (NM) states.

To this end, we calculated the phonon dispersion curves and phonon DOS of LFO in the FIM and NM states. Fig. 5 shows the calculated phonon dispersion curves along the high symmetry points of *X*, *R*, *M*, *Γ*, and *R* in the BZ, together with the total and atom-projected phonon DOS of LFO with a space group of *P*4_3_32 in the FIM and NM states. In the phonon dispersion curves, no anharmonic phonon modes with imaginary phonon energies were observed, indicating that these phases are dynamically stable. That is, it confirms that LFO is stabilized in the α-phase with the FIM or NM state under ambient conditions, as already reported in experiments.^28,43^ In the phonon dispersion curves of LFO with the NM state, there is a definite gap in the high frequency region around 80 meV unlike the FIM state. From the atom-projected phonon DOS, it is clear that the Fe atoms are responsible for lattice vibrations over the low frequency region while the O atoms have dominant contributions to the high frequency region for both FIM and NM states.

By post-processing the calculated phonon dispersions and phonon DOS, we finally evaluated the thermodynamic potential functions as functions of temperature and pressure for the FIM and NM states within QHA. Here we note that QHA can be valid up to the melting temperature of the crystal. By applying eqn (1), we calculated the Helmholtz free energies of LFO with the FIM and NM states as functions of volume at different temperatures (Fig. S9 and S10, ESI†). These obtained free energy–volume data were fitted to EOS at each temperature, yielding the equilibrium volume and bulk modulus. We therefore plotted the unit cell volumes (*V*) and cubic expansion coefficient (*α*) as functions of temperature ranging from 0 to 2000 K (Fig. S7, ESI†). We also provided the bulk moduli (*B*) and heat capacity (*C*_P_) evaluated at each temperature (Fig. S8, ESI†). With increasing temperature, *V* and *α* were found to increase while *B* and *C*_P_ decrease, which is in agreement with the common knowledge about thermal expansion of crystalline materials.

**Fig. 6:**
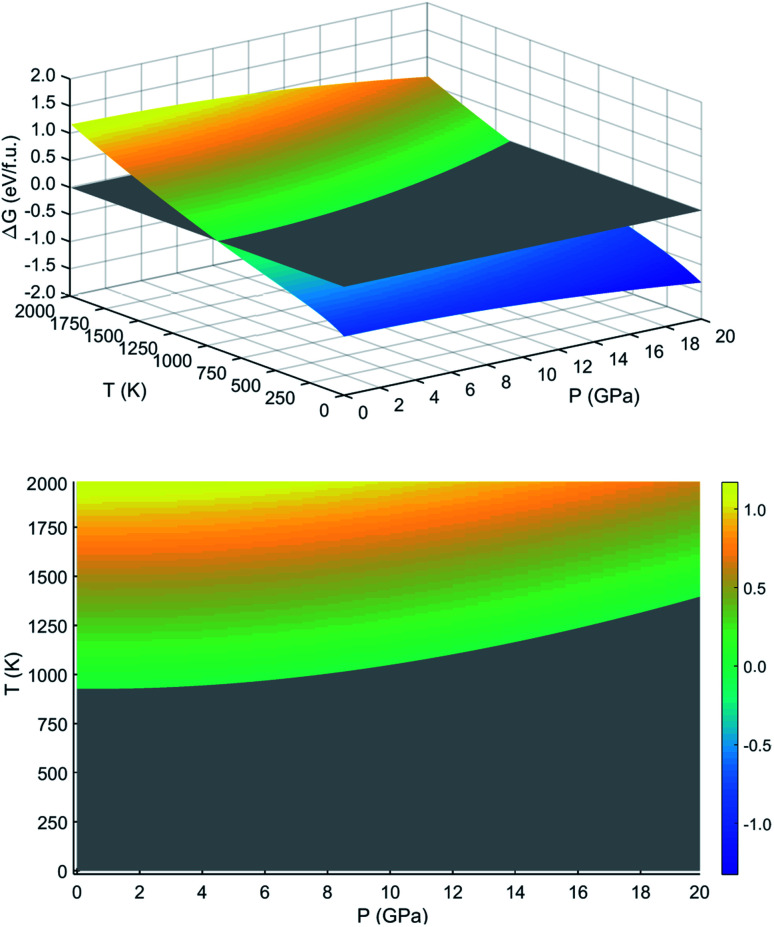
Gibbs free energy difference Δ*G* between the FIM and NM states of the ordered α-phase of LiFe_5_O_8_ with increasing temperature from 0 to 2000 K and increasing pressure from 0 to 20 GPa. The bottom panel displays the projection on the plane at Δ*G* = 0 (gray).

Fig. 6 shows the calculated Gibbs free energy differences between the FIM and NM states as a function of temperature and pressure. This can be regarded as a kind of *P*–*T* diagram for the thermodynamic stability of the FIM state against the NM state. From this *P*–*T* diagram, it was revealed that the FIM state in LFO is stable in the temperature range 0 to ∼925 K at atmospheric pressure (10^5^ Pa). After all, the Curie temperature *T*_C_ is ∼925 K under normal conditions, which is in agreement with the experimental value of 943 K.^15^ Furthermore, we demonstrated from the *P*–*T* diagram that *T*_C_ was gradually increased as pressure increased, and therefore, the ferrimagnetic state could remain stable at higher temperatures upon higher pressure.

## 4 Conclusions

In this work, we have systematically investigated the structural, electronic, optical, lattice vibrational properties and thermodynamic stability of lithium ferrite, LiFe_5_O_8_, using first-principles calculations. From the structural optimization, we revealed that the ordered α-phase (space group *P*4_3_32) with the ferrimagnetic spin configuration is energetically favourable among the crystalline phases with various magnetic configurations, in agreement with experimental observations. Using the DFT + *U* approach with *U* = 4 eV, we obtained a lattice constant of 8.34 Å, band gap energy for majority spin state of 1.99 eV, and total magnetization of 2.5 *μ*_B_ f.u.^−1^, also in good agreement with the available experimental data. From the calculation of light or electromagnetic wave absorption coefficient, LFO was expected to be suitable for applications of microwave devices. Our calculations for phonon dispersion revealed that LFO is stabilized in the ordered α-phase with the FIM or NM state due to no anharmonic phonon modes under ambient conditions. Finally, we evaluated the Gibbs free energy differences between the FIM and NM states at finite temperatures and pressures, and obtained the *P*–*T* diagram for thermodynamic stability of the FIM state against the NM state, demonstrating that the Curie temperature *T*_C_ is ∼925 K at the normal condition and gradually increased as pressure increases. Such a high magnetization and relatively high Curie temperature of LFO are desirable for permanent magnet applications where high operating temperatures and strong intensity of electromagnetic field are required.

## Author contributions

Su-Yong Kim and Kwang-Su Kim developed the original project, performed the calculations and drafted the first manuscript. Un-Gi Jong, Chung-Jin Kang and Song-Chol Ri assisted with the DFT calculations and the post-processing of calculation results, and contributed to useful discussions. Chol-Jun Yu supervised the work. All authors reviewed the manuscript.

## Conflicts of interest

There are no conflicts to declare.

## Supplementary Material

RA-012-D2RA01656G-s001
