# Supplementary material for: First-principles study on structural, electronic, magnetic and thermodynamic properties of lithium ferrite LiFe5O8
Source: RSC Adv. 2022 May 26;12(25):15973–9. doi: 10.1039/d2ra01656g (PMC9134028; doi:10.1039/d2ra01656g)
Supplement: RA-012-D2RA01656G-s001 [file RA-012-D2RA01656G-s001.pdf]

Electronic Supplementary Material (ESI) for RSC Advances.
This journal is © The Royal Society of Chemistry 2022

Supplementary Information – First-principles study on structural, electronic, magnetic and thermodynamic properties of lithium ferrite $LiFe_5O_8$

Su-Yong Kim[a], Kwang-Su Kim[b], Un-Gi Jong[a*], Chung-Jin Kang[a], Song-Chol Ri[b], and Chol-Jun Yu[a†]

[a] *Chair of Computational Materials Design (CMD), Faculty of Materials Science, Kim Il Sung University, Pyongyang, PO Box 76, Democratic People's Republic of Korea*

[b] *Institute of Functional Materials, Faculty of Materials Science, Kim Il Sung University, Pyongyang, PO Box 76, Democratic People's Republic of Korea.*

Table S1. Lattice constants (a), DFT total energies ($E_{\rm tot}$) and total magnetization ($M_{\rm tot}$) per formula unit (f.u.) for various magnetic ordering of $LiFe_5O_8$ with space groups of $P4_332$ and $Fd3m$. FOM, FIM and NM stand for the ferro-, ferri- and non-magnetic states, respectively.

Space group	Magnetic order	a (Å)	$E_{\rm tot}$ (eV/f.u.)	ΔE (eV/f.u.)	$M_{\rm tot}$ (μ_B/f.u.)
$P4_332$	FOM	8.29	-90.141	3.706	6.5
	FIM	8.32	-93.848	0.000	2.5
	NM	7.97	-93.641	0.206	0.0
$Fd3m$	FIM	8.27	-89.984	3.864	2.5
Exp.	FIM	8.33	–	–	2.5

*Un-Gi Jong, Email: ug.jong@ryongnamsan.edu.kp
†Chol-Jun Yu, Email: cj.yu@ryongnamsan.edu.kp

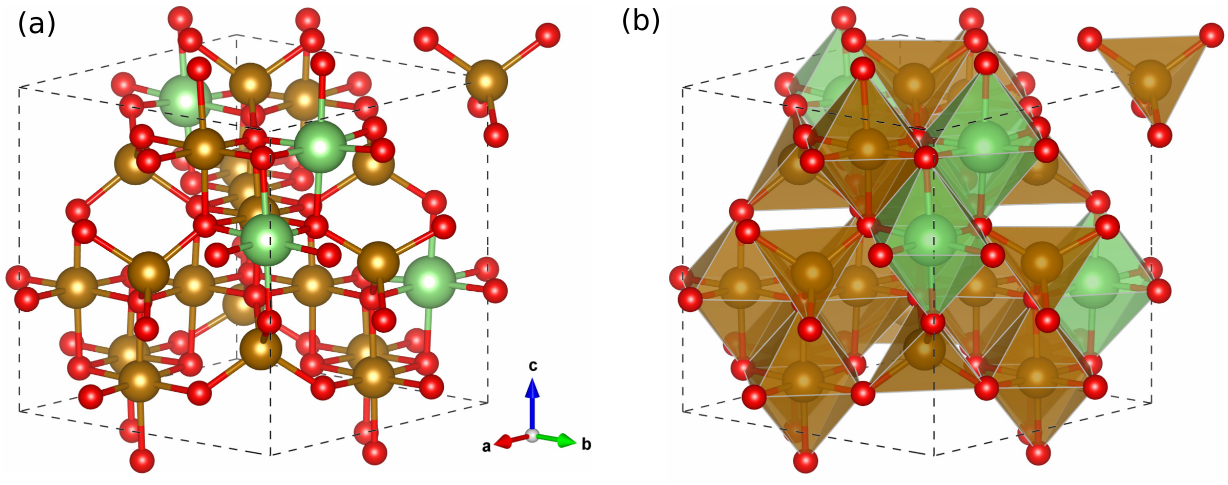

Figure S1. (a) Ball-and-stick view and (b) polyhedral view for crystalline structure of cubic $LiFe_5O_8$ with a space group of $Fd3m$. The brown, green and red balls represent Fe, Li and O atoms, respectively.

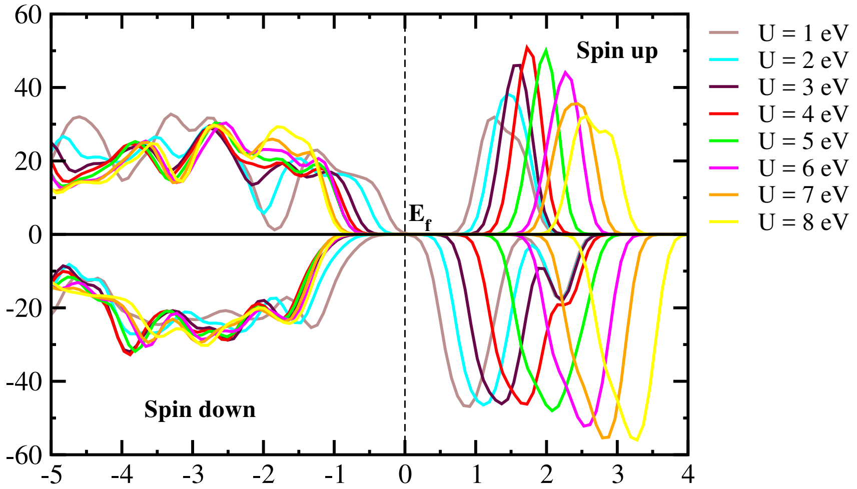

Figure S2. Total density of states for the spin up (positive) and down (negative) states calculated by DFT+U approach with gradually increasing values of on-site Coulomb interaction parameter U from 1 to 8 eV with a step of 1 eV in the ordered α-phase $LiFe_5O_8$ with the ferrimagnetic ordering.

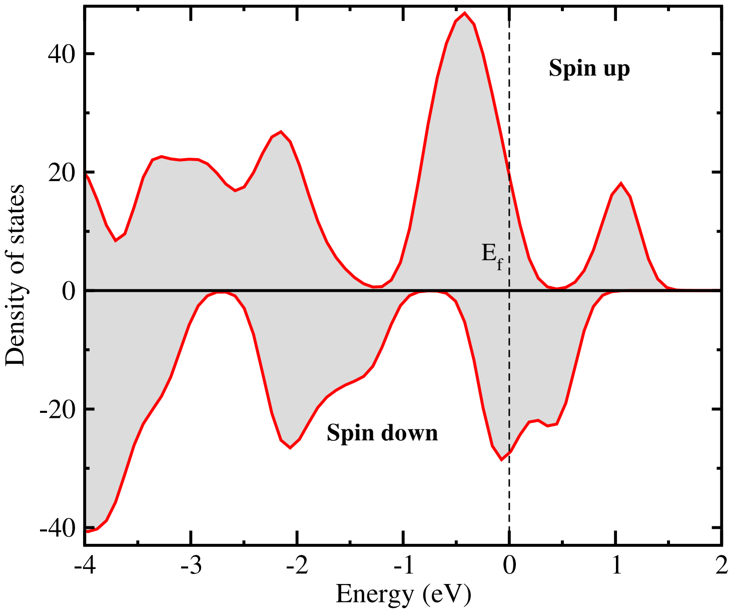

Figure S3. Total density of states for the spin up (positive) and down (negative) states calculated without considering the on-site Coulomb interaction for the ordered α-phase $LiFe_5O_8$ with the ferrimagnetic ordering.

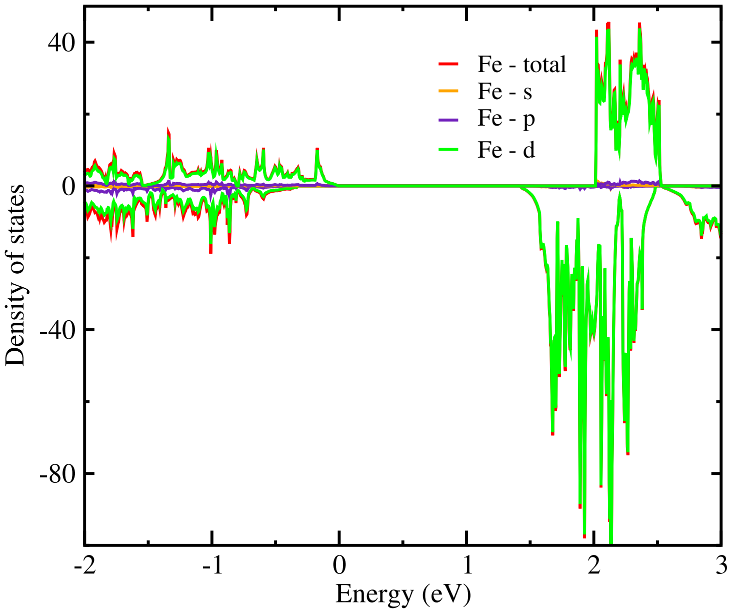

Figure S4. Orbital-projected density of states for the spin up (positive) and down (negative) states of Fe atoms calculated by the DFT+U approach with U = 4 eV for the ordered α-phase $LiFe_5O_8$ with the ferrimagnetic ordering.

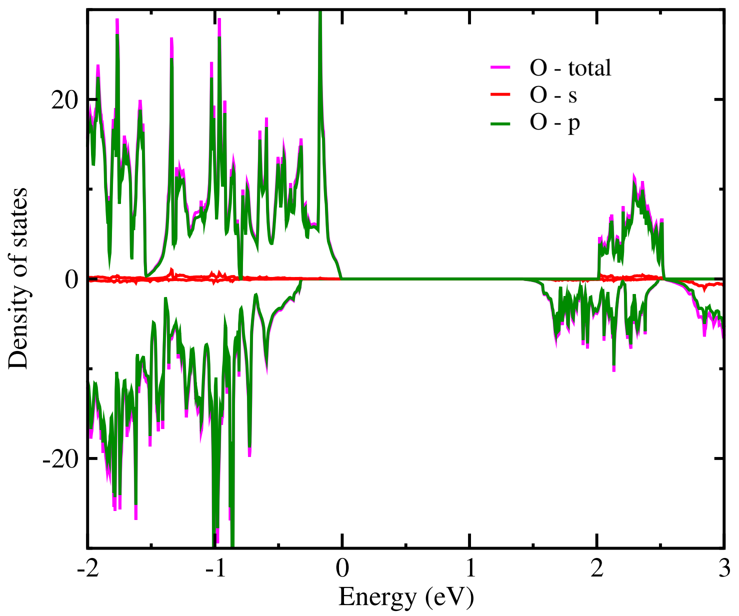

Figure S5. Orbital-projected density of states for the spin up (positive) and down (negative) states of O atoms calculated by DFT+U approach with U = 4 eV for the ordered α-phase $LiFe_5O_8$ with the ferrimagnetic ordering.

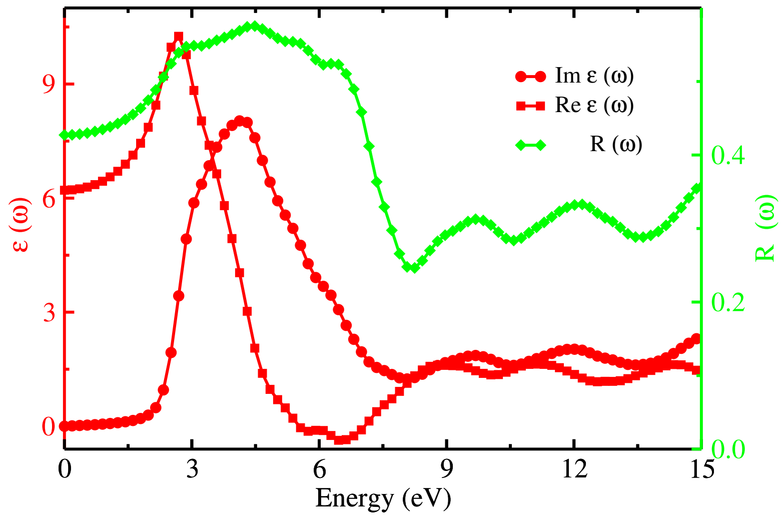

Figure S6. Frequency-dependent dielectric constant $\varepsilon(\omega)$ and reflectivity R(ω) calculated by DFT+U approach with U = 4 eV for the ordered α-phase $LiFe_5O_8$ with the ferrimagnetic ordering.

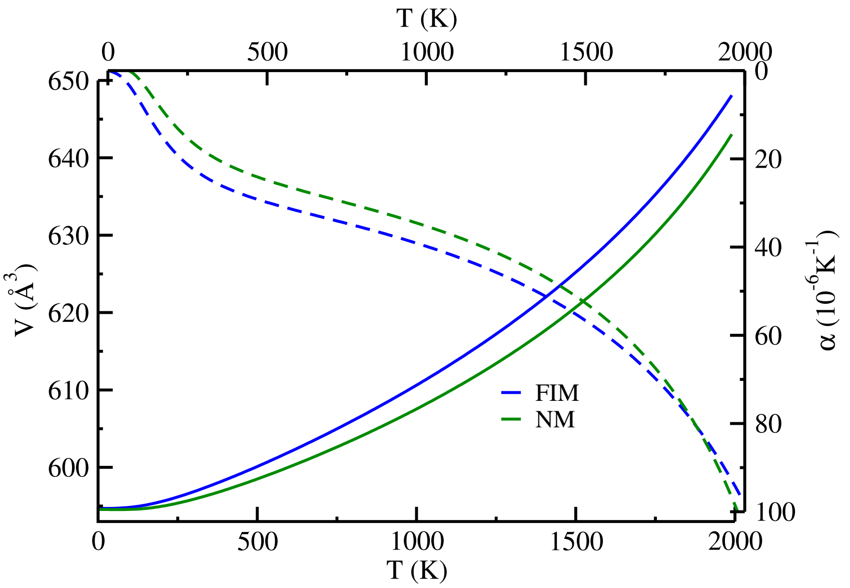

Figure S7. Unit cell volumes V (solid lines) and volume expansion coefficients (dashed lines) of cubic α-phase $LiFe_5O_8$ with the FIM and NM states as functions of temperature.

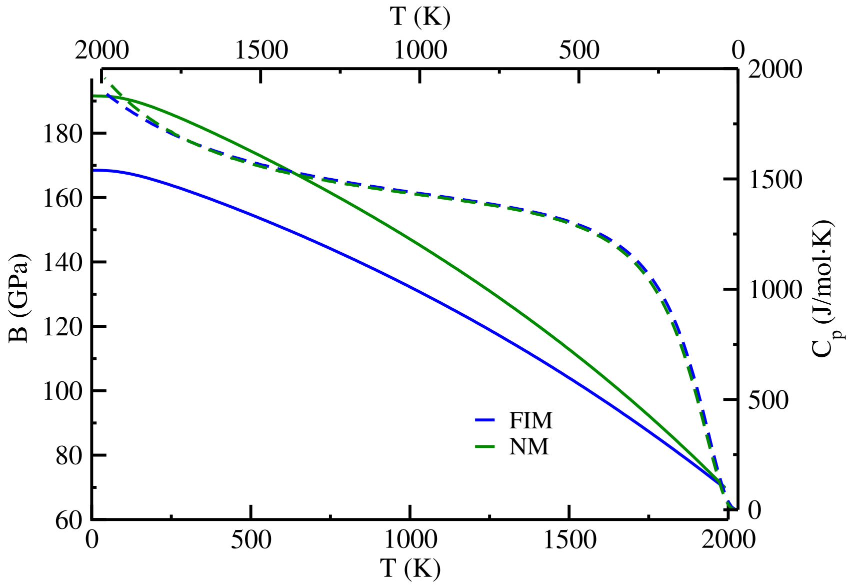

Figure S8. Bulk moduli B (solid lines) and heat capacities C_P (dashed lines) of cubic α-phase $LiFe_5O_8$ with the FIM and NM states as functions of temperature.

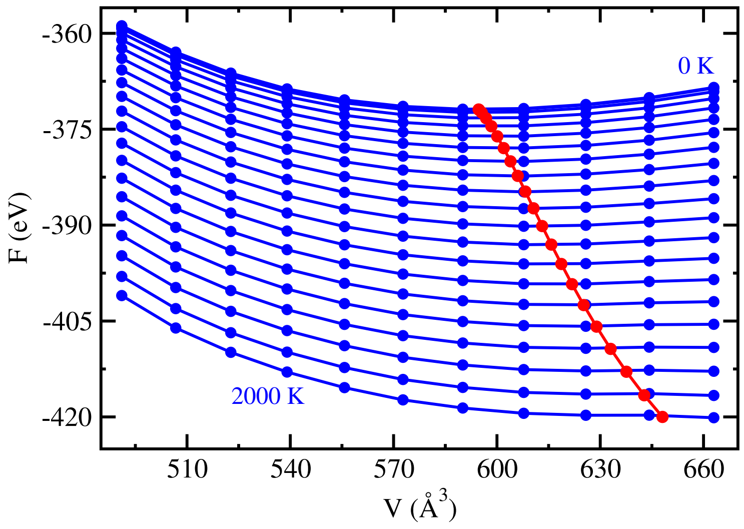

Figure S9. Helmholtz free energy curves (blue lines) as functions of unit cell volume at different temperatures for the FIM state. The red circles indicate equilibrium volumes at given temperature.

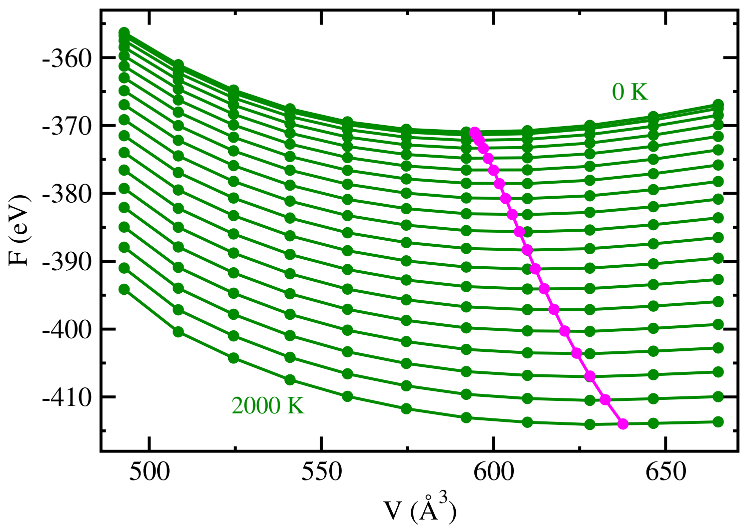

Figure S10. Helmholtz free energy curves (green lines) as functions of unit cell volume at different temperatures for the FIM state. The magenta circles indicate equilibrium volumes at given temperature.

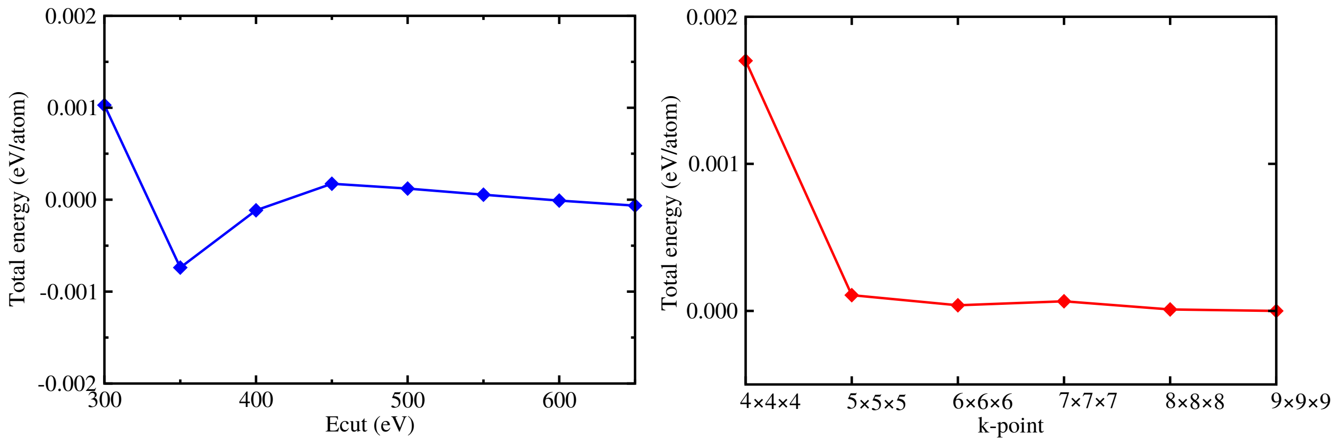

Figure S11. Total energy convergence tests according to the sizes of energy cutoff and *k*-point mesh for $LiFe_5O_8$.
